# *NF1*‐mutated melanoma tumors harbor distinct clinical and biological characteristics

**DOI:** 10.1002/1878-0261.12050

**Published:** 2017-03-24

**Authors:** Helena Cirenajwis, Martin Lauss, Henrik Ekedahl, Therese Törngren, Anders Kvist, Lao H. Saal, Håkan Olsson, Johan Staaf, Ana Carneiro, Christian Ingvar, Katja Harbst, Nicholas K. Hayward, Göran Jönsson

**Affiliations:** ^1^ Division of Oncology and Pathology Department of Clinical Sciences Lund University Sweden; ^2^ Division of Surgery Department of Clinical Sciences Lund University Sweden; ^3^ Department of Oncology Skåne University Hospital Lund University Sweden; ^4^ QIMR Berghofer Medical Research Institute Brisbane Australia

**Keywords:** *BRAF*, melanoma, *NF1*, *NRAS*

## Abstract

In general, melanoma can be considered as a UV‐driven disease with an aggressive metastatic course and high mutational load, with only few tumors (acral, mucosal, and uveal melanomas) not induced by sunlight and possessing a lower mutational load. The most commonly activated pathway in melanoma is the mitogen‐activated protein kinase (MAPK) pathway. However, the prognostic significance of mutational stratification is unclear and needs further investigation. Here, *in silico* we combined mutation data from 162 melanomas subjected to targeted deep sequencing with mutation data from three published studies. Tumors from 870 patients were grouped according to *BRAF*,*RAS*,*NF1* mutation or triple‐wild‐type status and correlated with tumor and patient characteristics. We found that the *NF1*‐mutated subtype had a higher mutational burden and strongest UV mutation signature. Searching for co‐occurring mutated genes revealed the RASopathy genes *PTPN11* and *RASA2*, as well as another RAS domain‐containing gene *RASSF2* enriched in the *NF1* subtype after adjustment for mutational burden. We found that a larger proportion of the *NF1*‐mutant tumors were from males and with older age at diagnosis. Importantly, we found an increased risk of death from melanoma (disease‐specific survival, DSS; HR, 1.9; 95% CI, 1.21–3.10; *P *= 0.046) and poor overall survival (OS; HR, 2.0; 95% CI, 1.28–2.98; *P *= 0.01) in the *NF1* subtype, which remained significant after adjustment for age, gender, and lesion type (DSS *P *= 0.03, OS *P *= 0.06, respectively). Melanoma genomic subtypes display different biological and clinical characteristics. The poor outcome observed in the *NF1* subtype highlights the need for improved characterization of this group.

**Abbreviations**
DSS: disease‐specific survival
FDR: false discovery rate
MAPK: mitogen‐activated protein kinase
NGS: next‐generation sequencing
OS: overall survival
TCGA: the Cancer Genome Atlas

## 1 Introduction

During recent years, next‐generation sequencing (NGS) has been introduced to the field of cancer medicine to identify patient‐ and tumor‐specific genetic alterations aiding in prognosis, confirmation of diagnosis, and guidance of therapeutic strategy. Genomic analyses have enabled the discovery of genetic subtypes in melanoma, which are reflected by specific aberrations in key molecular pathways associated with certain treatment modalities (Vidwans *et al*., 2011). The most commonly activated pathway in melanoma is the mitogen‐activated protein kinase (MAPK) pathway, often constitutively activated through mutations in the V600 codon of *BRAF* (in 35–50% of melanomas) and the Q61 codon of *NRAS* (10–25%) (Tsao *et al*., 2012). However, the prognostic significance of mutated *BRAF* and *NRAS* is unclear as contradictory findings have been reported (Ekedahl *et al*., 2013; Jakob *et al*., 2012; Rutkowski *et al*., 2014; Thomas *et al*., 2015). In contrast to most studies including MAPK inhibitor‐treated patients, Carlino *et al*. performed a retrospective study of advanced melanomas naïve to MAPK inhibitors and concluded that *BRAF* and *NRAS* mutation status did not influence survival from metastatic melanoma (Carlino *et al*., 2014).

More recently, a framework for genomic classification of melanoma has been proposed by the Cancer Genome Atlas network (TCGA) (Cancer Genome Atlas Network, 2015). The four subtypes have been defined based on the mutational pattern in *BRAF*,* RAS*,* NF1*, or none of these, the so‐called triple‐wild‐type group. *NF1* has been pinpointed as an important melanoma‐associated gene in previous studies. Hodis *et al*. found that tumors without recurrent mutations in either *BRAF* or *NRAS* had a significant enrichment of *NF1* mutations or alterations in *KIT* (Hodis *et al*., 2012). Furthermore, Krauthammer *et al*. identified a class of sun‐exposed melanomas with wild‐type *BRAF* and *NRAS* with few copy number aberrations, high mutational load, and inactivation of tumor suppressors, such as *NF1*,* TP53*,* ARID2*, and *PTPRK* (Krauthammer *et al*., 2012). Also, a report on whole‐exome sequencing identified *NF1* as the third most frequently mutated gene in melanoma after *BRAF* and *NRAS*, occasionally concurrently with other RASopathy gene mutations (Krauthammer *et al*., 2015). *NF1* is a tumor suppressor gene encoding a direct negative regulator of RAS (Bernards and Settleman, 2005), which cooperates with mutated BRAF in melanomagenesis by preventing oncogene‐induced senescence (Maertens *et al*., 2013).

While the option of targeted therapy is available for patients with *BRAF* V600‐mutant melanoma, patients with melanomas of the *RAS*,* NF1*, or triple‐wild‐type subtypes usually have no efficient therapeutic option besides immunotherapy. For *BRAF/NRAS* wild‐type tumors harboring *KIT* mutations in exons 11 and 13, imatinib may be an alternative (Guo *et al*., 2011; Hodi *et al*., 2013). For melanomas with *NF1* loss‐of‐function mutations or deletions, studies have shown that *NF1* ablation can be linked to decreased sensitivity and resistance to BRAF inhibitors both *in vitro* and *in vivo* (Maertens *et al*., 2013; Whittaker *et al*., 2013). In addition, preclinical studies have proposed sensitivity to MEK inhibition for *NF1*‐impaired melanomas (Nissan *et al*., 2014; Ranzani *et al*., 2015). In an attempt to identify therapeutic options for the *BRAF* wild‐type melanomas, there are now several clinical trials using mutational profiles for patient stratification (ClinicalTrials.gov identifier: NCT02645149, NCT02094872). Consequently, clinical mutation screening beyond *BRAF* and *NRAS* would be of significance in the clinical setting of melanoma.

In the present study, we performed integrated bioinformatics analyses of four datasets comprising a total of 870 independent tumors, in order to extensively characterize the mutational landscape of melanoma. Also, we assessed the clinical implication of *BRAF*,* RAS*,* NF1*, and triple‐wild‐type melanomas. Our findings showed that tumor mutational load varied within clinical variables such as gender, tumor type, age at diagnosis, and melanoma origin. We found a significant difference in survival outcome across the genomic subtypes, with the *NF1* subtype associated with poor survival in a cohort largely consisting of metastatic melanoma. The large size of the sample set enabled the identification of more subtle genetic aberrations converging on signaling pathways and mutational processes that may be important for melanoma development. Overall, our results suggest that mutations in key melanoma driver genes may predict tumor and patient phenotype.

## 2 Materials and methods

### 2.1 Clinical samples

The in‐house study (from here on called ‘Lund’) comprised 162 melanomas and patient‐matched clinical information. All samples were obtained at the Department of Surgery at Skåne University Hospital, Lund, Sweden. The majority of the samples (146/162) and the associated molecular data were used in a previous study with a focus on gene expression‐based analysis (Cirenajwis *et al*., 2015). The tumor cohort was retrospectively collected between 2000 and 2012. Overall, 95 patients (59%) were untreated and 67 patients (41%) were treated. Of the 67 cases, 13 patients received neoadjuvant treatment (nine chemotherapy and four immunotherapy). Only seven cases received targeted molecular therapy (four received BRAF inhibitor, two received imatinib, and one received sorafenib), two were treated with a vaccine, 24 cases were treated with immunotherapy (mainly interferon treatment), and 23 cases received chemotherapy. Treatment was initiated when patients had developed distant metastatic disease. The Lund study was approved by the Regional Ethics Committee at Lund University (Dnr. 191/2007 and 101/2013).

### 2.2 DNA extraction and analysis

In the Lund study, DNA libraries were prepared using Agilent SureSelect custom design approach comprising 1697 frequently mutated cancer‐associated genes selected based on information in the COSMIC database and from the literature (Harbst *et al*., 2014). Briefly, sequencing was performed on Illumina HiSeq 2000 (Illumina, Inc., San Diego, CA, USA) in a paired‐end mode to produce 2 × 101 bp reads. Reads were aligned using Novoalign (http://www.novocraft.com/products/novoalign/) and further processed using Picard (http://picard,sourceforge.net/) and the Genome Analysis Tool Kit (GATK) (DePristo *et al*., 2011). Somatic variants were called using VarScan2 (Koboldt *et al*., 2012).

### 2.3 Data analysis

We combined mutation data from the Lund study with mutation data from studies by Hodis *et al*. (2012), the TCGA project (Cancer Genome Atlas Network, 2015), and Krauthammer *et al*. (2015). In all external studies, the somatic mutation data in MAF format were provided and were downloaded from the supplementary section of each of the publications (Hodis *et al*., 2012; Krauthammer *et al*., 2015), whereas for the TCGA project, automated somatic calls were downloaded for 472 melanoma tumors from the TCGA data portal (frozen March 14, 2016) (Cancer Genome Atlas Network, 2015). Oncotator was used to annotate the effect on the protein level for identified somatic mutations (https://confluence.broadinstitute.org/display/CGATools/Oncotator). In the Lund data, we used VarScan2 ‘somatic’ to screen the 1697 genes for mutations (SNVs) in tumor–normal pairs. For somatic variant calling, minimum allowed coverage of 8 × in normal and 6 × in tumor was used. For a base position to be called mutated, there had to be a minimum of four mutant reads, and these had to constitute at least 10% of all reads at the base position. In addition, the variant allele frequency of the matched normal sample was allowed to be 3% at maximum. Sets of filtering steps are applied subsequently to ensure the quality of the calls. In case the requirements were not fulfilled, the base call remained to be wild‐type. We obtained a mean on target coverage of 198–594 reads. When analyzing the mutational load in the compiled cohort and when correcting for mutational burden in logistic regression models, we included SNVs (noncoding, synonymous and nonsynonymous mutations), whereas in the remaining analyses we focused on the nonsynonymous mutations, including missense, nonsense, nonstop, focal indels (both frame shift and in frame), and splice site SNVs.

**Figure 1:**
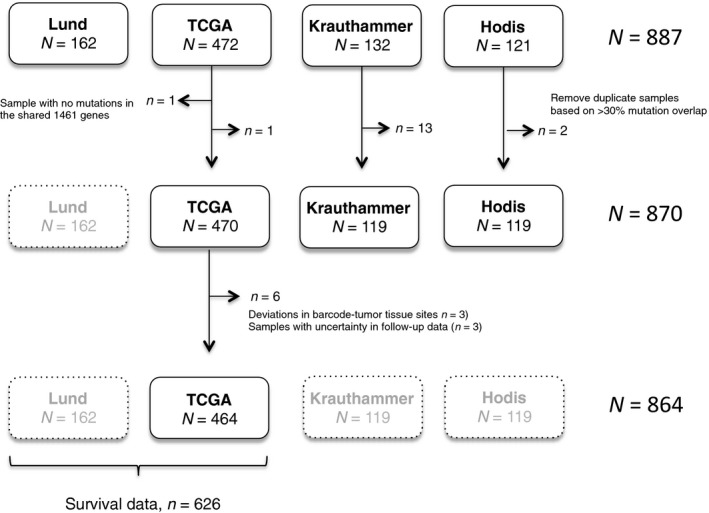
Flow‐chart describing the compilation of the cohort and detailed description.

All four combined studies comprised a large number of sequenced tumors ranging from 121 to 472 samples (only including samples with matched normal) (Fig. 1). Three studies provided whole‐exome sequencing data, whereas the Lund study comprised targeted deep sequencing of 1697 frequently mutated cancer‐associated genes. In total, 1461 genes were shared between the four datasets, and one sample was left out because it had no somatic mutations for any of these genes. Consequently, these shared genes were used in all downstream analyses. We then wanted to make sure that none of the samples in the Krauthammer *et al*. (11) and Hodis *et al*. (9) studies were included in the TCGA study and screened for samples that had > 30% agreement in mutations and removed 15 samples. In addition, a primary tumor was removed in one primary/metastasis pair in the TCGA data. In total, this gave a combined cohort of *N* = 870 melanomas (Fig. 1). We also collected available clinical data and removed six samples from the TCGA project due to uncertainty in tumor tissue site or in follow‐up data. Treatment information was available for 582 cases in the Lund and TCGA studies. In detail, 181 cases were treated during metastatic disease and 74 cases received chemotherapy, 73 cases received immunotherapy, 12 cases were vaccine‐treated, and only 17 cases received targeted therapy. A summary of the individual studies and the compiled cohort with clinical data (*n* = 864) is provided in Table S1. Mutation and clinical annotation files for the compiled cohort and the 1461 shared genes have been added as Supplementary Information (Tables S2 and S3).

### 2.4 Statistical analyses

Screening for significantly mutated genes was performed using the MutSigCV algorithm (Lawrence *et al*., 2013). Fisher's exact test was used to evaluate correlations between clinical variables in the total cohort, or stratified upon the genomic groups, and finally to further examine the prevalence of mutated genes or mutated signaling pathways across the genomic subtypes. Correction for multiple testing was performed using the *p.adjust* function with the ‘false discovery rate’ (FDR) setting in R. Moreover, logistic regression models were used to adjust for mutational load (all types of mutations). Specifically, a logistic regression was fitted for each gene with the dependent variable ‘gene mutation status’ and the predictors ‘genomic subtype’ and ‘sample total mutational load’. Wilcoxon or Kruskal–Wallis tests were used to compare the mutational load across the genomic subtypes or the clinical characteristics. All survival analyses were made using the *survival* package in R, where the four genomic subtypes were evaluated for their impact on overall survival (OS) and disease‐specific survival (DSS). Survival was estimated from when sample was surgically removed to last follow‐up or an event occurred. Survival analyses were further performed in metastases and primary tumors separately. *P*‐values were calculated based on a five‐year censoring of survival data from the time of biopsy. The *deconstructSigs* R package was used to analyze mutational signatures in the cohort (Alexandrov *et al*., 2013b). Mutational signatures were derived from the 1461 overlapping genes and included only samples with at least 50 somatic mutations.

## 3 Results

### 3.1 Mutational burden in clinical melanoma subgroups

A total of 1461 genes were shared between all four datasets and subsequently used in downstream analyses. Overall, the cohort included more male than female patients (61% and 39%, respectively), and the majority of tumors were from metastatic lesions (82%), while only 17% were from primary tumors. Only 2% of the tumors were non‐sun‐induced melanomas (acral or mucosal melanomas) (Table S1).

**Figure 2:**
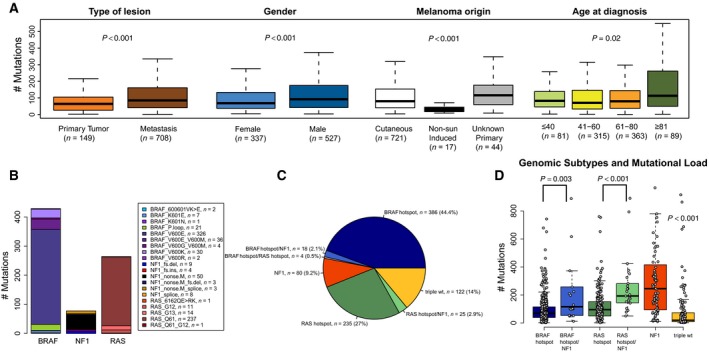
Mutational burden in association with clinical parameters in 864 melanoma tumors. (A) The total number of somatic mutations (coding and non‐coding) was determined for each patient and further correlated to clinical factors such as tumor type, melanoma origin, gender and age. (B–D) Genetic activation of the MAPK pathway in melanoma (*n *=* *870). A schematic overview of selected MAPK mutations in *BRAF*,*RAS* and *NF1* with each gene was analyzed separately with no consideration of cross gene co‐occurring events (B). The melanoma samples were further classified into mutational subtypes based on hotspot mutations in *BRAF* (affecting amino acid V600 and/or K601), *RAS* (Q61, G12, G13) or any non‐synonymous mutation in *NF1* (C), and correlated to mutational burden (D). Non‐parametric Kruskal–Wallis and Wilcoxon tests were used to calculate *P*‐values (A and D).

The total mutational load in the compiled cohort ranged from 1 to 3457 mutations per tumor, with a median of 80 mutations per tumor. A higher mutational burden was observed in metastases than in primary tumors (median: 85, range: 1–3457 versus median: 64, range: 2–1456, respectively; Kruskal–Wallis test, *P *<* *0.001; Fig. 2A). Tumors derived from male patients had a higher mutational burden than tumors from female patients (median: 90, range: 1–3457 versus median: 66, range: 2–1199, respectively; Wilcoxon test, *P *<* *0.001; Fig. 2A); when stratified upon tumor type, this difference was only significant for metastases (Kruskal–Wallis test, *P *=* *0.002). Moreover, there was a great variation in mutational load by site of tumor origin, with non‐sun‐induced tumors (acral lentiginous and mucosal, *n *=* *17) having a significantly lower mutational burden (median: 21, range: 6–145) than tumors with unknown primary (median: 114, range: 6–348) or cutaneous tumors (median: 78, range: 1–3457). Moreover, we found that older patients (> 81 years at diagnosis) had the highest mutational burden, whereas other age categories showed no difference (age ≤ 40 median 83 (range 3–394), age 41–60 median 71 (range 2–1199), age 61–80 median 80 (range 1–3457), and age ≥ 81 median 113 (range 2–1456), Kruskal–Wallis test, *P* = 0.02; Fig. 2A). Collectively, mutational load characterizes different clinical subgroups in melanoma.

### 3.2 Genetic activation of the MAPK pathway in melanoma

The majority of melanomas have an activated MAPK pathway, and recently, melanoma tumors were stratified into genomic subtypes according to mutations in the *BRAF*,* RAS*, or *NF1* genes (Cancer Genome Atlas Network, 2015). Of the 870 cases examined, 452 (52%) had nonsynonymous mutations in *BRAF*, and of those, 408 cases (90%) had a recurrent hotspot mutation at the V600 or K601 residues (Fig. 2B). Apart from hotspot mutations affecting the activation loop (A‐loop) in the enzymatic kinase domain of the BRAF protein, mutations in the phosphate‐binding loop (P‐loop) were identified. BRAF P‐loop mutations affecting amino acids 466‐471 were identified in 21 cases (5%, Fig. 2B). There was a mutually exclusive pattern between A‐loop and P‐loop mutations (Fisher's test, *P* < 0.001), suggesting that P‐loop mutations may be relevant in melanoma development. The *RAS* (*NRAS*,* KRAS*, and *HRAS*) genes had nonsynonymous mutations in 274 cases (31%), with 87% being Q61 mutations and 9% being G12/13 mutations (Fig. 2B). One case harbored concurrent *NRAS* Q61 and *KRAS* G12 mutations. The *NRAS* gene had nonsynonymous mutations in 255 cases (29%), of which 91% corresponded to Q61 mutations and 6% to G12/13 mutations. The *HRAS* gene had nonsynonymous mutations in eight cases (1%), of which 25% were Q61 mutations and 50% were G13 mutations. Finally, the *KRAS* gene had nonsynonymous mutations in 13 cases (1%), of which 31% were Q61 mutations and 46% were G12/13 mutations. With few exceptions, *BRAF* and *RAS* hotspot mutations were mutually exclusive (Fisher's exact test, *P *< 0.001). Nonsynonymous mutations in *NF1* were found in 123 cases (14%), and 77 of these (63%) were loss‐of‐function events (Fig. 2B). *BRAF* and *RAS* hotspot mutations were rarely found in tumors with nonsynonymous mutations in *NF1* (Fisher's exact test, *P* < 0.001 and *P *=* *0.008, respectively). Other members of the MAPK pathway such as *KIT*,* GNA11*, and *GNAQ* were mutated at low frequencies. Thirty‐two cases had nonsynonymous mutations in *KIT* (4%), with an enrichment of p.K642E (*n* = 7, 22%), p.V559A (*n* = 4, 12.5%), p.N822K (*n* = 2, 6%), p.L576P (*n* = 2, 6%), and p.W557R (co‐occurring with p.N822I, *n* = 1, 3%) mutations in the cohort. Only half of all tumors with *KIT* mutations did not harbor any of the hotspot mutations in *BRAF*,* RAS* genes or nonsynonymous mutation in *NF1*, and most of those cases (11/14) were enriched for the recurrent mutations mentioned above. Herein, two cases belonged to melanomas of acral lentiginous origin and comprised the p.K642E or p.N822K mutations, respectively. A minor fraction of the tumors in this cohort had mutations in *GNA11* (*n *= 17, 2%) and *GNAQ* (*n *= 15, 2%). *GNA11‐*mutated tumors had an enrichment of p.Q209L [*n* = 5 (one case with co‐occurring p.Q209H mutation), 24%] and p.R183C (*n* = 2, 12%) mutations. Six of the 17 *GNA11*‐mutated tumors did not harbor any of the hotspot mutations in *BRAF*,* RAS* genes or nonsynonymous mutation in *NF1*, and five of those cases harbored a p.Q209 mutation. Herein, two of the cases belonged to melanomas of uveal origin and comprised the p.Q209L mutation. *GNAQ‐*mutated tumors had an enrichment of p.Q209P (*n* = 3, 20%) and p.R183 alterations (p.R183 nonsense mutations, *n* = 2, 13%; p.R183Q, *n *= 1, 7%). Five of the 15 *GNAQ*‐mutated tumors did not harbor any of the hotspot mutations in *BRAF*,* RAS* genes or nonsynonymous mutation in *NF1*, and three of those cases had a p.Q209P mutation. However, we did not discern any non‐sun‐induced cases with nonsynonymous *GNAQ* mutations. The observed mutations in *GNA11/GNAQ* represent the most commonly reported mutations in the COSMIC database (http://cancer.sanger.ac.uk/cosmic). There seemed to be a mutually exclusive pattern between mutations in *KIT* and *GNA11/GNAQ*, with only three cases having co‐occurring mutations, although significance was not reached because of too few events. Of the 19 non‐sun‐induced melanomas in the cohort (uveal, *n *= 2; acral/mucosal, *n* = 17), only four cases contained recurrent mutations in *KIT* or *GNA11*.

Overall, we confirmed that mutations in the MAPK pathway occur mainly by constitutive active mutations in *BRAF* and *NRAS* or nonsynonymous mutations in the *NF1* gene. These mutations were found in a near to mutually exclusive fashion. Finally, other less frequently mutated melanoma genes (*KRAS*,* HRAS*,* KIT*,* GNAQ*,* GNA11*) were enriched in tumors wild‐type for *BRAF*,* NRAS*, and *NF1*.

### 3.3 Molecular characteristics of melanoma genomic subtypes

The 870 cases were stratified according to the genomic subtypes described previously (Cancer Genome Atlas Network, 2015): the *BRAF* subtype (*n* = 404, 46%), the *RAS* subtype (*n* = 260, 30%), the *NF1* subtype (*n* = 80, 9%), and the triple‐wild‐type subtype (*n* = 122, 14%) (Fig. 2C, Table 1). Notably, a small but significant fraction harbored a *BRAF* (*n* = 18, 2%) or *RAS* (*n* = 25, 3%) hotspot mutation along with a nonsynonymous mutation in *NF1* (Fig. 2C). Three of 18 cases with a *BRAF* hotspot mutation had a concurrent loss‐of‐function mutation in *NF1*, while 11 of 25 *RAS*‐mutant cases harbored a concurrent loss‐of‐function mutation in *NF1*. Only four cases had co‐occurring hotspot mutations in *BRAF* and *RAS* and were excluded from further analyses.

**Table 1:** Clinical characteristics and association with the four genomic subtypes

	Whole cohort (*N *=* *864)	BRAF hotspot (*N *=* *403)	NF1 (*N *=* *79)	RAS hotspot (*N *=* *259)	Triple‐wt (*N *=* *119)	*P*‐value^a^,^b^
Tumor type						
Primary	149 (17)	82 (20)	16 (20)	27 (10)	24 (20)	0.003
Metastasis	708 (82)	319 (80)	63 (80)	231 (89)	91 (76)	
NA	7 (1)	2 (< 1)	0 (0)	1 (< 1)	4 (3)	
Melanoma origin						
Cutaneous	721 (83)	340 (84)	69 (87)	214 (83)	95 (80)	0.006^c^
Unknown primary	44 (5)	23 (6)	0 (0)	15 (6)	5 (4)	
Non‐sun induced^d^	17 (2)	4 (1)	4 (5)	3 (1)	6 (5)	
Other^e^	4 (< 1)	1 (< 1)	1 (1.3)	0 (0)	2 (2)	
NA	78 (9)	35 (9)	5 (6)	27 (10)	11 (9)	
Gender						
Female	337 (39)	161 (40)	21 (27)	99 (38)	54 (45)	0.06
Male	527 (61)	242 (60)	58 (73)	160 (62)	65 (55)	
Age, mean (years)	61	56	72	64	66	< 0.001

a Not including data for four co‐occurring RAS BRAF hotspot mutants.

b By Fisher's exact test, except for age at submitted specimen (one‐way ANOVA).

c Only including cutaneous, non‐sun induced and melanomas of unknown origin.

d Including mucosal and acral lentiginous melanomas.

e Including uveal and tumors from other anatomical sites.

Tumors in the *NF1* subtype had a higher mutational burden (median: 246, range: 10–3457) as compared to the RAS (median: 95; range: 1–760), *BRAF* (median: 69; range: 2–1158), or triple‐wild‐type (median: 19.5; range: 2–916) groups (Kruskal–Wallis test, *n* = 870, *P *<* *0.001) (Fig. 2D). Analysis of mutational burden in cases with co‐occurring *BRAF* hotspot and *NF1* nonsynonymous mutations (median: 116, range: 12–1199) revealed that such cases had an increased mutational burden as compared to *BRAF* mutations alone (Wilcoxon test, *P *=* *0.003, Fig. 2D). A similar pattern was found in the RAS‐mutant group with cases harboring co‐occurring *RAS* hotspot and *NF1* nonsynonymous mutations (median: 193, range: 50–891) having an increased mutational burden compared to the tumors harboring *RAS* mutations alone (Wilcoxon test, *P *<* *0.001, Fig. 2D). However, in this study, cases with co‐occurring mutations in *NF1* and hotspot mutations in *BRAF or RAS* were thus assigned to the *BRAF* and *RAS* genomic subtypes, respectively.

Next, we investigated associations of any gene mutation (ignoring *BRAF*,* RAS*, and *NF1* genes) with the genomic subtypes. First, we searched for significantly mutated genes in the cohort through the MutSigCV algorithm, finding six significantly mutated genes (*BRAF*,* NRAS*,* TP53*,* CDKN2A*,* PTEN*, and *CTNNB1*, FDR ≤ 0.01). Here, we found that *PTEN* mutations were significantly enriched in the *BRAF* subtype (FDR = 0.008), whereas mutations in *TP53* (FDR < 0.001) and *CDKN2A* (FDR = 0.03) were enriched in the *NF1* subtype. Second, we screened the entire gene set for associations between gene mutations and genomic subtypes. Due to an extensive background mutational rate in the *NF1* subtype, more than half of all genes (*n* = 825) investigated were found more frequently mutated in this group as compared to the other genomic groups. In order to find putative driver genes in the *NF1* subtype, a logistic regression model was built to analyze the mutational status for each gene while adjusting for mutational burden (all types of mutations). Thirty‐six mutated genes (FDR < 0.05) were enriched across the *NF1* mutants in a mutation frequency range of 5–36% (Table S4) including the previously described RASopathy genes *PTPN11* and *RASA2* (11), as well as another RAS domain‐containing gene *RASSF2*. Applying the MutSigCV software on *NF1*‐mutated tumors exclusively further validated these results. Although no statistically significant gene was identified, the top ranked genes agreed with our logistic regression model (Table S5). Furthermore, for mutated genes normally related to non‐sun‐induced melanomas (*KIT*,* GNA11*,* GNAQ*), we found that *KIT* and *GNA11* were enriched in the triple‐wild‐type group (FDR < 0.001 or 0.01, respectively). Within the subset of tumors comprising nonsynonymous mutations in *KIT*,* GNA11*, and/or *GNAQ* (*n* = 59), cases with recurrent mutations (highlighted in previous section) revealed a strong correlation with the triple‐wild‐type group (Fisher's test, *n* = 59, *P* < 0.001) and a small enrichment within non‐sun‐induced tumors [Fisher's test, *n* = 51 (cutaneous versus non‐sun‐induced cases), *P *=* *0.04]. Thus, the four non‐sun‐induced cases with recurrent mutations in *KIT* or *GNA11* were all classified as triple‐wild‐type. However, the 15 remaining sun‐induced tumors did not belong to a specific genomic group. In conclusion, molecular characterization of the genomic subtypes reveals several subtype‐specific characteristics and gene mutations.

### 3.4 Somatic mutations in key molecular pathways in cancer

To date, approximately 140 cancer driver genes have been identified in sequencing studies of major tumor types. These genes can be further organized into 12 signaling pathways or three core cellular processes (Vogelstein *et al*., 2013). We addressed the frequency and pattern of nonsynonymous mutations in these genes and corresponding pathways in our compiled melanoma cohort. In total, data from 79 genes were available for analysis; however, only genes that exclusively belonged to one of the pathways were included, leaving 64 genes for analysis (Table 2). As expected, the RAS pathway was mutated in the majority of melanomas (89%) and the MAPK pathway (*GNA11* and *GNAQ*) in 4% of melanomas, displaying a mutually exclusive pattern with mutations in the RAS pathway (*P* = 0.001). Other pathways frequently mutated in melanoma included chromatin modification (47%) and DNA damage control (21%). When adjusting for mutational burden in a logistic regression model with the *BRAF* hotspot tumors as the reference group, we found the Hedgehog pathway enriched in the *NF1* subtype, the cell cycle/apoptosis pathway less mutated in the triple‐wild‐type group, the chromatin modification pathway enriched in the *RAS* subtype, the MAPK pathway (*GNA11* and *GNAQ*) enriched in the *RAS* subtype and triple‐wild‐type tumors, whereas the latter two groups harbored less mutations in the PI3K pathway (Table 2). Collectively, some but not all major cancer pathways are frequently mutated in melanoma.

**Table 2:** Vogelstein's pathways and mutation prevalence in the cohort (*n* = 870)

Cancer cell signaling pathways/processes	Unique pathway genes	Mutated pathway^d^ (prevalence in cohort), %	*P*‐value^e^^,^ a (difference across the genomic groups)
Cell fate			
NOTCH	FBXW7, NOTCH1, NOTCH2	13	Not sign
HH	PTCH1, SMO	5	< 0.001^b^
APC	APC, AXIN1, CDH1, CTNNB1, FAM123B, HNF1A, NF2	23	Not sign
Chromatin modification	ARID1A, ARID1B, ATRX, DNMT1, DNMT3A, EXH2, KDM6A, MEN1, MLL2, MLL3, PBRM1, SETD2, SMARCA4, SMARCB1	47	0.03^c^
Transcriptional regulation	AR, GATA3, RUNX1	7	Not sign
Genome maintenance			
DNA damage control	ATM, BAP1, BRCA1, BRCA2, MLH1, MSH2, MSH6	21	Not sign
Cell survival			
TGF‐β	ACVR1B, SMAD4)	2	Not sign
MAPK	GNA11, GNAQ	4	0.04^d^
STAT	JAK1, JAK2, JAK3	8	Not sign
PI3K	AKT1, PIK3CA, PIK3R1, PTEN, TSC1	16	0.002^d^
RAS	BRAF, CIC, HRAS, KRAS, NF1, NRAS, PTPN11	89	Not sign
Cell cycle/Apoptosis	ABL1, BCL2, CASP8, CDC73, CDKN2A, CYLD, RB1, TRAF7	24	0.008^e^

Non‐synonymous mutations in any of the genes.

Not including data for four co‐occurring RAS BRAF hotspot mutants.

a Adjusting for mutational burden (all mutation types) in a logistic regression model with BRAF hotspot tumors as the reference group.

b The pathway is more frequently mutated in the NF1 genomic subtype.

c The pathway is more frequently mutated in the RAS hotspot genomic subtype.

d The pathway is more frequently mutated in the RAS hotspot and triple‐wild‐type genomic subtypes.

e The pathway is less frequently mutated in the triple‐wild‐type genomic subtype.

### 3.5 Mutational signatures in clinical and genomic subgroups of melanoma

**Figure 3:**
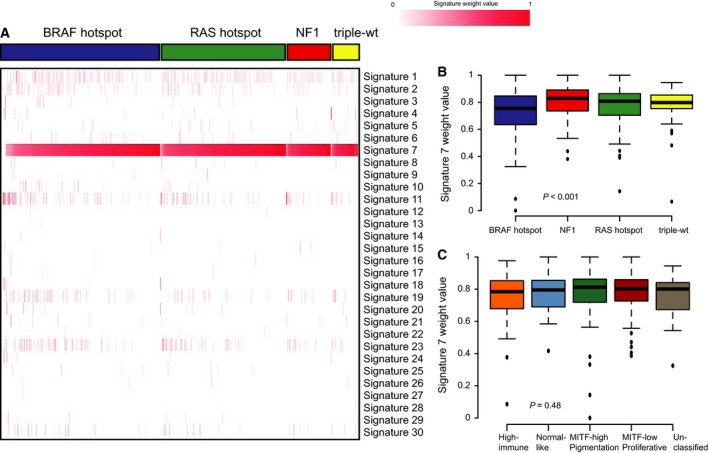
Mutational processes in melanoma. (A) Heatmap of signature weight values with samples ordered according to genomic subtypes and signature 7 (UV) activity. (B) Boxplot of signature 7 (UV) weight values across the four genomic subtypes. (C) Boxplot of signature 7 (UV) weight values across gene expression subtypes (excluding unclassified cases). Non‐parametric Kruskal–Wallis tests were used to calculate *P*‐values (B,C).

Recently, a range of distinct mutational processes was defined using whole‐exome or whole‐genome sequencing data (Alexandrov *et al*., 2013a,b). Some of these processes are induced by external mutagens such as UV light and smoking. We used the compiled data and excluded samples with fewer than 50 somatic mutations due to statistical power, leaving us with 513 tumors, and used the R package *deconstructSigs* to derive the impact of the 30 mutational signatures put forward by Alexandrov *et al*. (2013a). As expected, we found signature 7, which is associated with UV exposure, to have the highest signature weight (Fig. 3A). Interestingly, we observed that there was a range (range 0–1, median 0.79) of weight values for signature 7. Only 27 cases had a value < 0.5 and only two cases had a weight value of zero. The latter had the highest value for signature 11, which exhibits mutational patterns resembling that of alkylating agent exposure. Most likely, these patients were treated with dacarbazine or temozolomide prior to when their biopsies were taken. Indeed, one of these patients was included in the Lund study and had received temozolomide prior to surgical removal consistent with the mutational signature. Next, we analyzed associations between mutational signatures and clinical features. We found no association between mutational signatures and gender, type of tumor, or age at diagnosis (*P *>* *0.05, Wilcoxon and Kruskal–Wallis test). A trend to significance was observed for age at diagnosis and the UV signature (*P *=* *0.02, Kruskal–Wallis test); however, after correction for multiple testing, significance was lost. Next, we determined the association between mutational signatures and mutational groups or gene expression subtypes as described previously (Jonsson *et al*., 2010). Tumors belonging to the *BRAF* hotspot‐mutant group had significantly lower association with the UV mutational signature, while *NF1*‐mutant tumors had stronger association with the UV signature (*P *<* *0.001, Kruskal–Wallis test), although we did not detect any difference with regard to gene expression subtypes (Fig. 3B,C). Overall, this suggests that the UV‐derived mutation signature is the dominating signature in melanoma, with only subtle differences based on driver gene mutations.

### 3.6 Clinical significance of genomic subtypes

When analyzing the genomic subtypes individually, the proportion of males was significantly higher in the *NF1* subtype as compared to the *BRAF* or triple‐wild‐type groups (Fisher's exact test, *P *=* *0.03 or 0.01, respectively). Patients with *BRAF*‐mutant tumors were generally younger (mean: 56 years; range: 20–90) than patients with *NF1*‐mutant tumors (median: 72 years; range: 41–92) (one‐way ANOVA test, *P* < 0.001, Table 1). Moreover, we also found an increased prevalence of metastatic lesions in the *RAS* group when compared to the other genomic subtypes (Fisher's exact test, *P *=* *0.003, Table 1). Overall, this suggests that there are distinct clinical features associated with the mutational subtypes.

**Figure 4:**
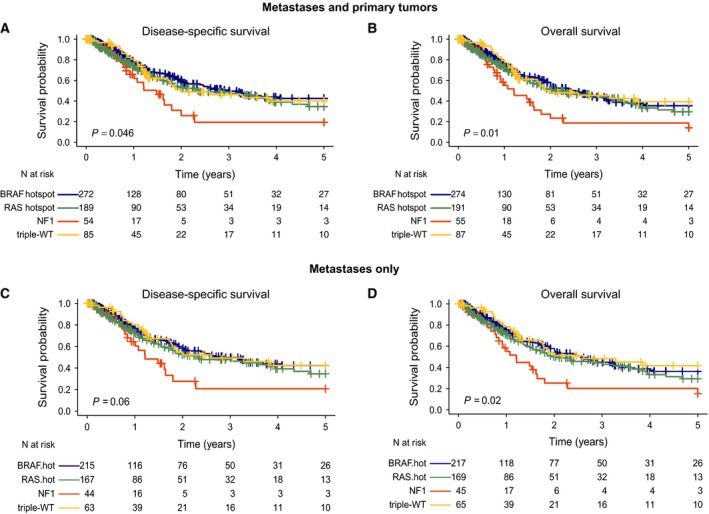
Five‐year survival analysis of melanomas stratified by the mutational subtypes using the Kaplan–Meier and log‐rank tests to determine (A, C) disease specific survival (DSS) and (B, D) overall survival (OS) in all tumors (A, B) and metastases only (C, D). Survival differences between the genomic groups were estimated using Kaplan–Meier analysis. *P*‐values have been calculated using the log‐rank test.

Next, we analyzed the association between the genomic subtypes and the survival outcome in the combined Lund and TCGA datasets (*n* = 626 patients). An increased risk of death from melanoma (five‐year disease‐specific survival, DSS; HR, 1.9; 95% CI, 1.21–3.10; *P *=* *0.046) and poor OS (HR, 2.0; 95% CI, 1.28–2.98; *P *=* *0.01) were observed in the *NF1* subtype compared to the reference group, *BRAF* (Fig. 4A,B). There was no significant difference between the other genomic subtypes (*P *>* *0.05). When adjusted for age, gender, and tumor type in a multivariable Cox regression model, with the *BRAF* group as the reference group, the *NF1* subtype still had the worst DSS (*P* = 0.03) and OS (*P* = 0.06). To investigate whether the observed difference was biased due to cohort composition, we analyzed the two studies separately and found the same trend in both cohorts (Fig. S1). Although the cohort consisted of mainly metastases, we performed a subgroup survival analysis with metastases and primary tumors separately. Statistical significance remained for OS and borderline significance for DSS when analyzing metastases exclusively (*P *=* *0.02 and *P *=* *0.06, respectively, log‐rank test, Fig. 4C,D). When adjusting for gender and age at diagnosis in multivariate Cox regression model, OS and DSS *P*‐values were borderline significant (*P *=* *0.09 and *P *=* *0.06, respectively). In primary tumors, exclusively no difference in survival difference was observed (*P *>* *0.05, log‐rank test). Finally, we analyzed survival outcome in treated and untreated patients separately. Although we found tumors of the NF1 subtype having worst outcome in both treated and untreated cases, significance was not reached in untreated cases (*P *>* *0.05, log‐rank test), while borderline significance was obtained in treated cases (*P *=* *0.06 OS and DSS, log‐rank test). As the *NF1* subtype appeared to have the worst survival outcome across all the four genomic subtypes, we also wanted to investigate whether patients with co‐occurring hotspot mutations in *BRAF* or *RAS* and nonsynonymous mutations in *NF1* had an impaired survival outcome as compared to the *BRAF* or *RAS* mutants alone. Of the 18 cases with co‐occurring hotspot mutation in *BRAF* and nonsynonymous mutation in *NF1*, only one patient had an event (OS or DSS), and thus, this mutational group could not be included in the survival estimate. However, the mutational group with co‐occurring hotspot mutations in *RAS* and nonsynonymous mutation in *NF1* had more events (12/25 cases) and showed a tendency of poor OS (HR, 1.6; 95% CI, 0.89–3.05; *P *=* *0.1) and DSS (HR, 1.5; 95% CI, 0.76–2.89; *P *=* *0.2) as compared to the reference group, *RAS* (Fig. S2). Collectively, these results highlight that *NF1* mutation may harbor prognostic information in the metastatic setting of melanoma.

## 4 Discussion

The significance of somatic mutation screening in the treatment‐predictive setting of melanoma is well established. Herein, we analyzed the clinical significance of mutation profiles and subtypes in a large cohort of melanoma tumors. Importantly, we found *NF1*‐mutated melanomas to harbor distinct biological characteristics and to be associated with poor survival outcome, suggesting that further characterization of these melanomas is required. The question of a clinically or biologically relevant melanoma classification based on molecular tumor features is a long‐standing problem. Several reports have suggested classification based on mutation status of key genes in the MAPK signaling pathway and/or sun exposure pattern (Curtin *et al*., 2005; Krauthammer *et al*., 2012). The present study provides extensive characterization of genomic subtypes based on mutations in *BRAF*,* RAS*, and *NF1* by using a large dataset comprising mutation data for 1461 genes in 864 clinically annotated melanomas. *BRAF*‐subtype tumors were typically found in younger patients (Cancer Genome Atlas Network, 2015). These tumors were enriched in *PTEN* mutations, confirming frequent co‐occurrence of *BRAF* and *PTEN* mutations in melanoma, as shown previously (Jonsson *et al*., 2007; Tsao *et al*., 2012). Somatic *NF1* alterations in melanoma were discovered in the early 1990s (Andersen *et al*., 1993; Johnson *et al*., 1993). We showed that the *NF1* subtype is a distinct biological and clinical entity, characterized by a high burden of somatic mutations, and typically prevalent in older male patients. We further demonstrated that tumors from the *NF1* subtype had a stronger correlation with UV mutagenesis, and the BRAF subtype a weaker correlation, as compared to the other groups. Interestingly, significant differences in tumor mutational load between men and women have recently been reported (Gupta *et al*., 2015). Due to the high mutational load in the *NF1* tumors, the identification of significant genetic aberrations in the *NF1* subtype is challenging. Indeed, most of the differentially mutated genes, cancer driver genes, and pathways identified in the present study had the highest mutation frequency in the *NF1* subtype as compared to the other genomic subtypes. Herein, we confirmed that mutations in the RASopathy genes *RASA2* and *PTPN11* were enriched in the *NF1* subtype (Krauthammer *et al*., 2015) and also identified *RASSF2*, a RAS domain‐containing gene, as enriched in the *NF1* subtype. These results further support that *NF1* cooperates with other RASopathy genes in melanomagenesis.

Although the division of melanoma by MAPK mutation status is biologically relevant and predictive in MAPK inhibitor therapy, controversy exists regarding the prognostic significance of such classification (Carlino *et al*., 2014; Ekedahl *et al*., 2013).

Neither could the TCGA study report any significant difference in postaccession survival across the four genomic subtypes (Cancer Genome Atlas Network, 2015). In this study, mainly due to increased statistical power, we demonstrate that *NF1* tumors have significantly worse DSS and OS as compared to the other genomic subtypes, even after adjustment for age, gender, and tumor type. Stratification on whether a primary tumor or metastasis was analyzed demonstrated that the most pronounced difference was observed in the metastatic setting. Further stratification on whether patients received systemic therapy or not showed the most extensive difference in survival outcome in treated patients. In all, this suggests that the prognostic significance of harboring *NF1* mutation may have a greater impact in the most advanced stages of metastatic melanoma although additional studies in larger cohorts with distant metastatic melanoma patients are needed. Similar results have earlier been indicated for *BRAF*‐mutant melanoma (Long *et al*., 2011). On the other hand, having any of the other genomic subtypes (*BRAF*,* RAS*, or triple‐wild‐type) did not translate into direct prognostic value in our study. In addition, *NF1* aberrations have been linked to more adverse outcomes in other cancer types such as breast cancer and head and neck squamous cell carcinoma (Lenarduzzi *et al*., 2013; Ogata *et al*., 2001). While being an adverse prognostic marker, a nonsynonymous *NF1* mutation can be a favorable treatment‐predictive marker, by virtue of its association with increased mutational load. In particular, tumors with high mutational burden (or deficiency in the DNA mismatch repair pathway leading to such an increase) have recently been shown to respond better to immune checkpoint blockade agents (Le *et al*., 2015; McGranahan *et al*., 2016; Rizvi *et al*., 2015; Van Allen *et al*., 2015). Furthermore, *NF1*‐mutant melanomas have been found to be dependent on MAPK signaling and to respond to inhibitors targeting key players of this pathway (MEK, ERK) (Maertens *et al*., 2013; Nissan *et al*., 2014; Whittaker *et al*., 2013). However, Ranzani and colleagues also claim that most *BRAF*/*NRAS* wild‐type melanomas are highly sensitive to MEK inhibition irrespectively of the NF1 protein level (Ranzani *et al*., 2015). This may shed some light on the triple‐wild‐type group and those melanomas not amenable to KIT inhibitory treatment. In addition, *NF1* mutations may also play role in the intrinsic and acquired resistance to RAF inhibition in melanoma (Whittaker *et al*., 2013). In this cohort, we found a subset of *BRAF‐*mutated tumors with co‐occurring nonsynonymous mutations in *NF1*. However, as we did not focus on MAPK therapeutics in this study, we can only speculate whether or not these tumors would have an impaired treatment response.

In summary, we confirmed that melanomas can be divided into four genomic subtypes based on recurrent mutations in the MAPK pathway. These groups represent distinct biological and clinical entities, with the *NF1* genomic subtype showing distinct features. The *NF1*‐mutated subtype has more mutations overall, possibly due to a UV signature and, although this should increase an immune response in patients, they show worse survival. This could be due to RASopathy genes and other crucial genes being hit by mutations more often. It remains to be clarified whether NF1‐subtype patients could benefit from immunotherapy more than other subgroup patients because they should show many neoantigens.

## Author contributions

HC, NKH, and GJ conceived the study. HC and GJ drafted the manuscript. HC, TT, and KH performed laboratory analyses. HC, ML, AK, LHS, KH, and JS performed bioinformatic analyses. HE, AC, HO, and CI collected clinical information. HC, ML, HE, TT, AK, LHS, HO, JS, AC, CI, KH, NKH, and GJ read and approved the final manuscript.

## Supporting information


**Fig. S1.** Five‐year survival analysis of melanomas stratified by the mutational subtypes using the Kaplan‐Meier estimator to determine (A) overall survival (OS) or (B) disease specific survival (DSS) in the ‘Lund’ or ‘TCGA’ datasets separately.
**Fig. S2.** Five‐year survival analysis of melanomas stratified by either hotspot mutations in *(N/H/K)RAS* alone or co‐occurrence with non‐synonymous mutations in *NF1* using the Kaplan–Meier estimator to determine (A) overall survival (OS) or (B) disease specific survival (DSS).
**Table S1.** Clinical characteristics of 864 melanoma patients and their tumors.
**Table S4.** Thirty‐six putative driver genes in the *NF1* genomic subtype.
**Table S5.** Recurrently mutated genes in NF1‐mutated cases using MutSigCV.

**Table S2**. Clinical annotations of the cohort.

**Table S3.** Mutation data of the shared 1461 genes across the entire cohort.
