# Supplementary material for: NF1‐mutated melanoma tumors harbor distinct clinical and biological characteristics
Source: Mol Oncol. 2017 Mar 24;11(4):438–51. doi: 10.1002/1878-0261.12050 (PMC5527484; doi:10.1002/1878-0261.12050)

NF1 mutated melanoma tumors harbor clinical and biological characteristics

Cirenajwis et al.

SUPPLEMENTARY IFORMATION

SUPPLEMENTARY TABLES

Supplementary Table 1. Clinical characteristics of 864 melanoma patients and their tumors.

	**Whole cohort (*N*=864)**	**Hodis et al. (*N*=119)**	**Krauthammer et al. (*N*=119)**	**TCGA (*N*=464)**	**Lund (*N*=162)**
Tumor type					
Primary	149 ()	15 (13)	28 (24)	100 (22)	6 (4)
Metastasis	708 ()	103 (87)	91 (76)	362 (78)	152 (94)
NA	6 (1)	1 (<1)	0 (0)	2 (<1)	4 (2)
Melanoma origin					
Cutaneous	721 (83)	93 (78)	106 (89)	429 (92)	93 (57)
Unknown primary	44 (5)	10 (8)	13(11)	0 (0)	21 (13)
Non-sun induced[1]	17 (2)	7 (6)	0 (0)	0 (0)	10 (6)
Other[2]	4 (<1)	1 (<1)	0 (0)	0 (0)	3 (2)
NA	78 (9)	8 (7)	0 (0)	35 (8)	35 (22)
Gender					
Female	337 (39)	53 (45)	43 (36)	179 (39)	62 (38)
Male	527 (61)	66 (55)	76 (64)	285 (61)	100 (62)
Age, mean (range)	61 (17-94)	52 (17-87)	68 (41-94)	61 (20-90)	63 (25-90)

1 Including mucosal and acral lentiginous melanomas.
2 Including uveal and tumors from other anatomical sites.

Supplementary Table 4. Thirty-six putative driver genes in the *NF1* genomic subtype.

Gene	BRAF.hot n^{mut} (%)	NF1.any n^{mut} (%)	RAS.hot n^{mut} (%)	Triple-wt n^{mut} (%)	P-value*
ABCB6	2 (0.5)	5 (6.2)	2 (0.8)	0 (0)	0.047
ABHD12B	2 (0.5)	9 (11.2)	2 (0.8)	1 (0.8)	0.003
CD44	4 (1)	7 (8.8)	2 (0.8)	1 (0.8)	0.009
CUBN	32 (7.9)	29 (36.2)	20 (7.7)	4 (3.3)	0.005
DLK1	5 (1.2)	8 (10)	4 (1.5)	1 (0.8)	0.044
ELF5	4 (1)	7 (8.8)	4 (1.5)	0 (0)	0.030
FBXO38	7 (1.7)	10 (12.5)	11 (4.2)	1 (0.8)	0.019
GPRC5B	3 (0.7)	6 (7.5)	4 (1.5)	0 (0)	0.046
HM13	1 (0.2)	5 (6.2)	2 (0.8)	1 (0.8)	0.027
KRT76	13 (3.2)	15 (18.8)	9 (3.5)	4 (3.3)	0.047
MAGEC2	15 (3.7)	20 (25)	14 (5.4)	5 (4.1)	0.034
MAK	6 (1.5)	13 (16.2)	7 (2.7)	2 (1.6)	0.036
MAPK13	3 (0.7)	7 (8.8)	5 (1.9)	2 (1.6)	0.030
MKL1	4 (1)	10 (12.5)	8 (3.1)	2 (1.6)	0.044
MMP14	1 (0.2)	5 (6.2)	2 (0.8)	0 (0)	0.030
MMP27	12 (3)	14 (17.5)	7 (2.7)	1 (0.8)	0.016
NRCAM	11 (2.7)	15 (18.8)	5 (1.9)	2 (1.6)	0.025
NUDCD1	3 (0.7)	7 (8.8)	2 (0.8)	0 (0)	0.044
OTOF	33 (8.2)	27 (33.8)	30 (11.5)	5 (4.1)	0.034
PIK3R5	13 (3.2)	14 (17.5)	8 (3.1)	2 (1.6)	0.007
PKNOX1	1 (0.2)	7 (8.8)	1 (0.4)	0 (0)	0.009
PRKCB	16 (4)	18 (22.5)	13 (5)	4 (3.3)	0.003
PTH2R	15 (3.7)	14 (17.5)	10 (3.8)	0 (0)	0.033
PTPN11	4 (1)	11 (13.8)	5 (1.9)	1 (0.8)	0.001
PTPRS	10 (2.5)	18 (22.5)	10 (3.8)	3 (2.5)	0.016
RASA2	11 (2.7)	18 (22.5)	5 (1.9)	3 (2.5)	0.001
RASSF2	4 (1)	9 (11.2)	3 (1.2)	2 (1.6)	0.009
RNF213	23 (5.7)	23 (28.7)	16 (6.2)	4 (3.3)	0.030
SMO	4 (1)	10 (12.5)	4 (1.5)	2 (1.6)	0.019
SMYD3	2 (0.5)	5 (6.2)	2 (0.8)	0 (0)	0.027
STAT4	18 (4.5)	19 (23.8)	8 (3.1)	5 (4.1)	0.040
TET1	14 (3.5)	22 (27.5)	21 (8.1)	6 (4.9)	0.016
TREM1	11 (2.7)	8 (10)	2 (0.8)	0 (0)	0.040
TSC1	6 (1.5)	11 (13.8)	2 (0.8)	2 (1.6)	0.009
WFDC1	1 (0.2)	4 (5)	2 (0.8)	1 (0.8)	0.048
ZNF25	2 (0.5)	8 (10)	2 (0.8)	0 (0)	0.004

* P values were obtained from a logistic regression model for mutational status (non-synonymous mutations) across the genomic subtypes while adjusting for mutational burden (non-coding and coding mutations). *P*-values have been corrected for multiple testing.

Supplementary Table 5. Recurrently mutated genes in NF1-mutated cases using MutSigCV. Top 10 genes of 1439 tested genes. Genes marked in red were also among the 36 genes found by logistic regression to be more frequently mutated in NF1-mutated cases.

Gene	Nonsilent mutations (n)	Silent mutations (n)	*P*	q	Rank NF1 mut	Rank NF1 wt
PTPN11	15	0	5.15E-03	1	1	1021

CDKN2A	19	2	3.87E-02	1	2	5
GPRC5B	6	3	8.80E-02	1	3	1304
GPR84	8	2	1.50E-01	1	4	17
ITK	28	4	2.16E-01	1	5	1320
RASSF2	9	1	2.23E-01	1	6	70
PPP1R3C	5	0	2.26E-01	1	7	147
PTEN	6	0	2.40E-01	1	8	4
ABHD12B	9	2	2.41E-01	1	9	569
NFIA	7	2	2.79E-01	1	10	220

LEGENDS FOR SUPPLEMENTARY FIGURES

Supplementary Figure 1

Five-year survival analysis of melanomas stratified by the mutational subtypes using the Kaplan-Meier estimator to determine (A) overall survival (OS) or (B) disease specific survival (DSS) in the "Lund" or "TCGA" datasets separately. Survival differences between the genomic groups were estimated using Kaplan-Meier analysis. P-values have been calculated using the log-rank test.

Supplementary Figure 2

Five-year survival analysis of melanomas stratified by either hotspot mutations in *(N/H/K)RAS* alone or co-occurrence with non-synonymous mutations in *NF1* using the Kaplan-Meier estimator to determine (A) overall survival (OS) or (B) disease specific survival (DSS). Survival differences between the genomic groups were estimated using Kaplan-Meier analysis. P-values have been calculated using the log-rank test.

Supplementary Figure 1

A)

Lund+TCGA (n=607)

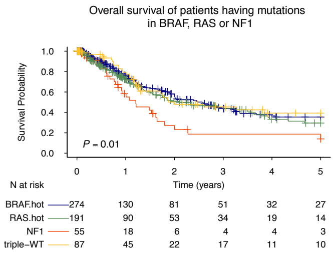

Lund data (n=160)

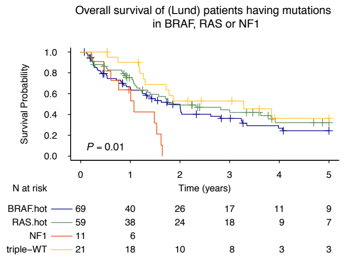

TCGA data (n=447)

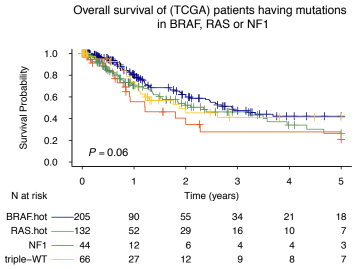

B)

Lund+TCGA (n=600)

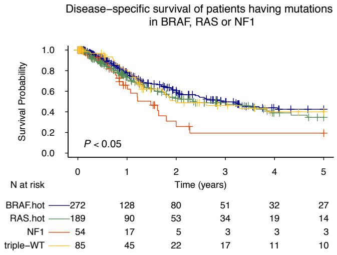

Lund data (n=160)

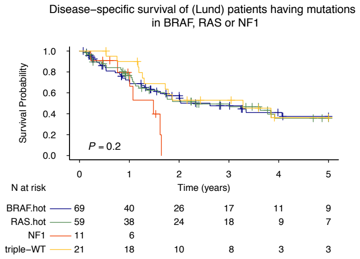

TCGA data (n=440)

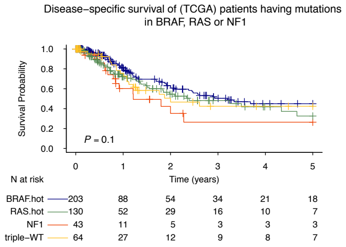

Supplementary Figure 2

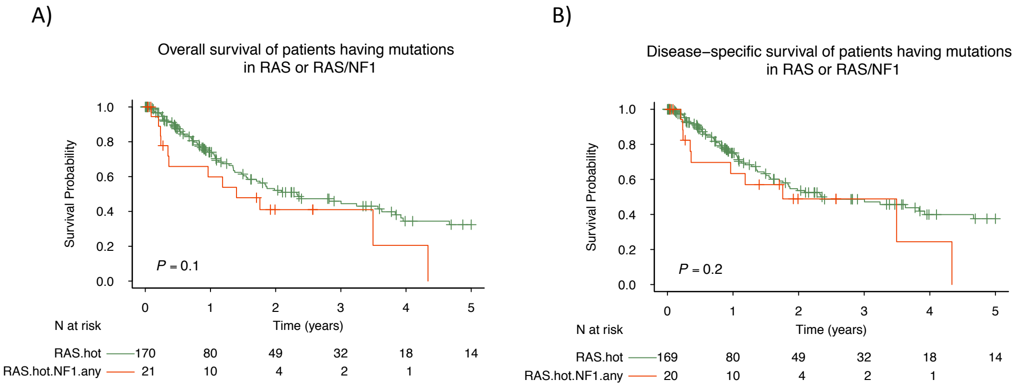

Supplement: Supplementary file 1 — Fig. S1. Five‐year survival analysis of melanomas stratified by the mutational subtypes using the Kaplan‐Meier estimator to determine (A) overall survival (OS) or (B) disease specific survival (DSS) in the ‘Lund’ or ‘TCGA’ datasets separately. Fig. S2. Five‐year survival analysis of melanomas stratified by either hotspot mutations in (N/H/K)RAS alone or co‐occurrence with non‐synonymous mutations in NF1 using the Kaplan–Meier estimator to determine (A) overall survival (OS) or (B) disease specific survival (DSS). Table S1. Clinical characteristics of 864 melanoma patients and their tumors. Table S4. Thirty‐six putative driver genes in the NF1 genomic subtype. Table S5. Recurrently mutated genes in NF1‐mutated cases using MutSigCV. [file MOL2-11-438-s001.pdf]
